# Aberrant CDK4 Amplification in Refractory Rhabdomyosarcoma as Identified by Genomic Profiling

**DOI:** 10.1038/srep03623

**Published:** 2014-01-10

**Authors:** Silvia Park, Jeeyun Lee, In-Gu Do, Jiryeon Jang, Kyoohyoung Rho, Seonjoo Ahn, Lira Maruja, Sung Joo Kim, Kyoung-Mee Kim, Mao Mao, Ensel Oh, Yu Jin Kim, Jhingook Kim, Yoon-La Choi

**Affiliations:** 1 Department of Medicine, Division of Hematology-Oncology, Samsung Medical Center, Sungkyunkwan University School of Medicine, Seoul, Korea; 2 Department of Pathology, Samsung Medical Center, Sungkyunkwan University School of Medicine, Seoul, Korea; 3 Korean Bioinformation Center (KOBIC), KRIBB, Daejeon 305-806, Korea; 4 Pfizer Oncology, 10724 Science Center Dr, San Diego, CA 92121, USA; 5 Department of Surgery, Samsung Medical Center, Sungkyunkwan University School of Medicine, Seoul, Korea; 6 Department of Thoracic Surgery, Samsung Medical Center, Sungkyunkwan University School of Medicine, Seoul, Korea; 7 Laboratory of Cancer Genomics and Molecular Pathology, Samsung Biomedical Research Institute, Samsung Medical Center, Seoul, Korea

## Abstract

Rhabdomyosarcoma (RMS) is the most commonly occurring type of soft tissue tumor in children. However, it is rare in adults, and therefore, very little is known about the most appropriate treatment strategy for adult RMS patients. We performed genomic analysis of RMS cells derived from a 27-year-old male patient whose disease was refractory to treatment. A peritoneal seeding nodule from the primary tumor, pleural metastases, malignant pleural effusion, and ascites obtained during disease progression, were analyzed. Whole exome sequencing revealed 23 candidate variants, and 10 of 23 mutations were validated by Sanger sequencing. Three of 10 mutations were present in both primary and metastatic tumors, and 3 mutations were detected only in metastatic specimens. Comparative genomic hybridization array analysis revealed prominent amplification in the 12q13–14 region, and more specifically, the *CDK4* proto-oncogene was highly amplified. ALK overexpression was observed at both protein and RNA levels. However, an ALK fusion assay using NanoString technology failed to show any ALK rearrangements. Little genetic heterogeneity was observed between primary and metastatic RMS cells. We propose that *CDK4*, located at 12q14, is a potential target for drug development for RMS treatment.

Rhabdomyosarcoma (RMS) is the most commonly occurring type of soft tissue tumor in children^1^, but it is less common in adults, accounting for only 2–5% of all soft tissue sarcomas^2^. Given the rarity of this disease, very little information is available on the most appropriate treatment strategy for adult RMS patients. Patients with unresectable or metastatic RMS have an extremely low cure rate and a poor prognosis^3^,^4^. A substantial improvement in survival has been achieved with the introduction of intensive chemotherapy regimens, which are usually based on pediatric oncology clinical trials on RMS^5^,^6^,^7^,^8^,^9^. However, survival rates for patients with metastatic disease remain disappointing, and the prognosis is dismal in patients with a poor response to salvage chemotherapy^3^,^10^. Thus, identification of novel therapeutic targets in RMS is urgently needed in order to improve treatment outcomes for this aggressive type of tumor.

Major histologic subtypes of RMS include embryonal RMS (ERMS) and alveolar RMS (ARMS)^11^. Despite advances in therapy, patients with the ARMS histological variant of RMS have a 5-year survival of less than 30%. ARMS presents with distinctive chromosomal translocations that result in specific fusion gene products, the most prevalent of which are PAX3–FOXO1 (55%) and PAX7–FOXO1 (22%)^12^. Reciprocal translocation of chromosomes 2 and 13 results in a *PAX3-FKHR* fusion gene in ARMS, which fuses the region of the gene encoding the DNA-binding domain of the transcription factor PAX3 with that encoding the transactivation domain of the transcription factor FKHR in-frame (3, 4). However, at least 25% of ARMS cases lack such translocations, suggesting that ARMS is not a single disease, but a heterogeneous group of conditions with a common phenotype. Moreover, studies on the gene expression profile of RMS have proposed new molecular classifications^13^ and have revealed that a specific gene expression signature potentially determines tumor behavior as well as treatment outcome^14^,^15^,^16^. *ALK* is one of the targets of interest, given that *ALK* alterations are relatively common in RMS, although the function of its gene product remains unknown^17^.

Here, we report the clinical application of genomic profiling in identifying potential novel genetic mutations in patients with relapsed and chemotherapy-refractory alveolar RMS.

## Results

### Case presentation

**Figure 1:**
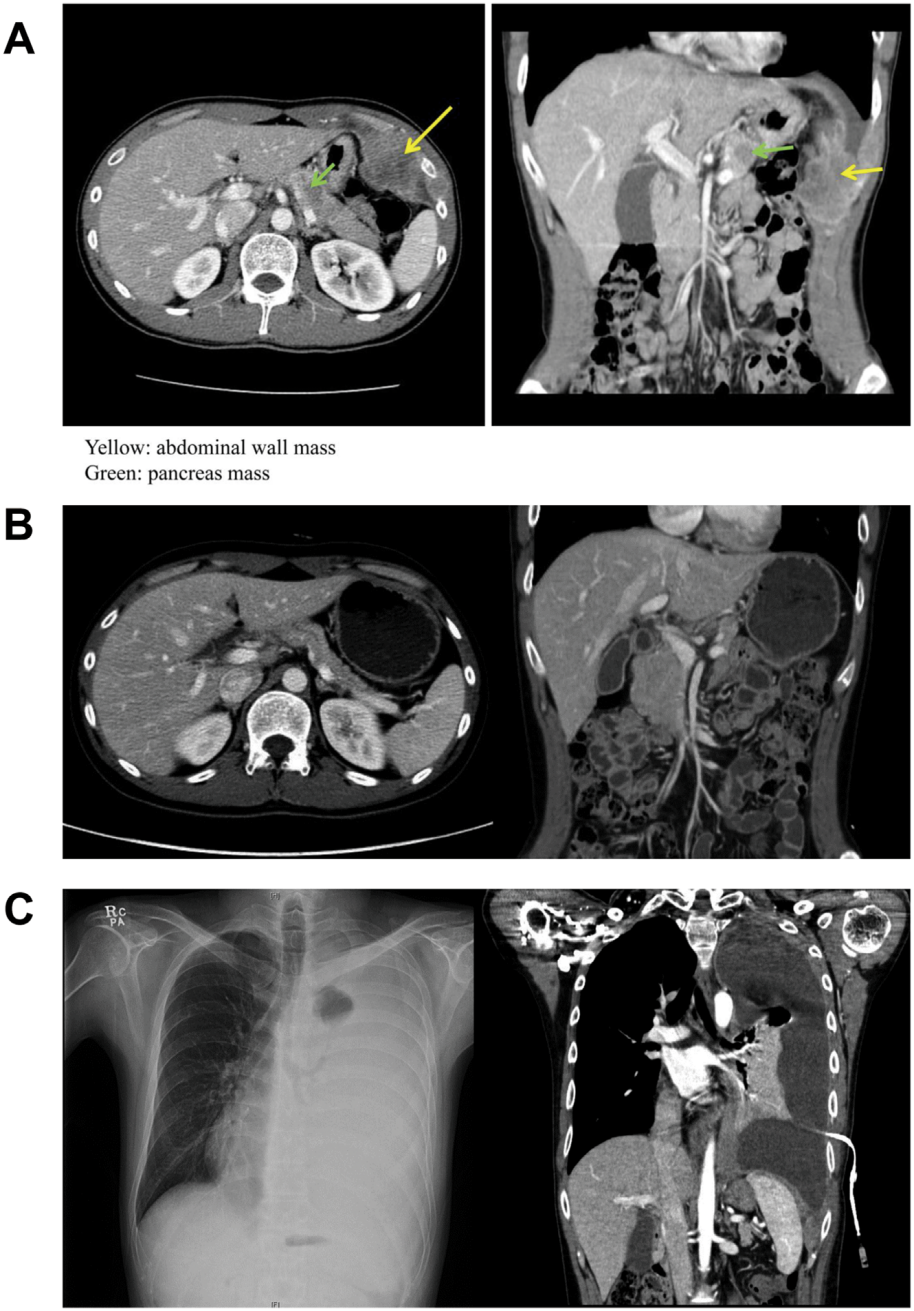
Computed tomography (CT) findings during the course of the disease. (A). Abdominal wall mass (yellow) and pancreas mass (green) at the time of initial diagnosis. (B). After 1^st^-line chemotherapy, the disease virtually disappeared. (C). A massive amount of left pleural effusion was seen after salvage chemotherapy.

A 27-year-old man presented with a complaint of left upper quadrant abdominal pain that had lasted for 3 years. Computed tomography (CT) and positron emission tomography (PET) scans showed multiple malignant masses, involving the pancreas and left upper abdominal wall, and pleural seeding was also noted (Fig. 1A). Pathological examination of the abdominal wall mass showed thin fibrous septae lined by small round blue cells in an alveolar growth or solid pattern; the cells appeared to lack cohesion and had hyperchromatic nuclei and scant cytoplasm. The tumor cells were diffusely positive for CD99, desmin, and WT1 and showed scattered focal positivity for cytokeratin. Ki-67 staining revealed high proliferative activity of the tumor cells. The *FKHR* break-apart fluorescence in situ hybridization (FISH) showed separate green and red signals, confirming *FKHR* rearrangement (Supplementary Fig. S1).

Based on the histology, immunohistochemistry (IHC), and FISH results, ARMS was diagnosed, and alternating cycles of vincristine, doxorubicin, and cyclophosphamide (VDC) and ifosfamide and etoposide (IE) were administered every 3 weeks.

After completing a 1-year course of cytotoxic chemotherapy, the patient achieved near complete remission, with disappearance of the multiple masses and pleural seeding (Fig. 1B). On the basis of a tumor board discussion involving a multi-modality team for sarcoma, the residual peritoneal seeding nodules were surgically resected. At the time of surgery, the resected seeding nodules were snap frozen and immediately stored at −80°C for molecular analysis. The pathologic examination of the resected peritoneal seeding nodules verified the diagnosis of ARMS.

Postoperative follow-up abdominal pelvis CT and chest CT demonstrated no evidence of malignancy. However, 3 months after surgical resection, the patient was found to have developed a chest wall mass of approximately 2 cm in size. Salvage etoposide, ifosfamide, and cisplatin (VIP) chemotherapy was administered and initially elicited a partially positive response. However, soon afterward, the patient developed rapidly progressive disease with a massive amount of left pleural effusion after the 5th cycle of VIP (Fig. 1C). Because dyspnea was caused by rapidly increasing pleural effusion, talc pleurodesis and pleural biopsy were performed through video-assisted thoracoscopic surgery (VATS). At this time, the patient agreed to full genetic testing using the tissue specimens to identify potential molecular targets (sample #2).

In addition, malignant cells from the pleural effusion were cultured and stored at −80°C for molecular analysis and *in vitro* drug sensitivity tests (sample #3). However, the patient developed malignant ascites (sample #4) immediately after pleurodesis, and 4 cycles of paclitaxel and ifosfamide were administered. The patient developed cord compression with paraplegia, experienced continued disease progression, and died several weeks later.

### Genomic profiling and somatic mutation

DNA from the primary tumor (sample #1) was sequenced, revealing 23 candidate variants: *SCN1B*, *PPP1R3A*, *GRID2*, *APBA2*, *ZNF142*, *ZYG11A*, *RBFOX1*, *TCF7L1*, *NARF*, *KIAA0182*, *TEX13B*, *MUC2*, *LRRC3*, *GRHL3*, *MUC16*, *TTR*, *UBA1*, *FEN1*, *ELAC2*, *NBEAL1*, *DSCAML1*, *PCDHA4*, and *POLR3C* (Table 1). Only the *MUC16* mutation was detected in blood, with 3.4% allele frequency. Amino acid substitutions were predicted to arise from some of the point mutations in each gene, and these in turn were predicted to have a substantial phenotypic effect based on the SIFT score (a SIFT score of 0 indicates a deleterious effect, a score ≤ 0.05 indicates a damaging effect, and a score > 0.05 suggests that the substitution can be tolerated). These protein mutations were also predicted to have considerable functional impact based on the FI score as determined by Mutation Assessor (http://mutationassessor.org) (an FI score ≤ 0.8 is considered neutral; 0.8 < FI score ≤ 1.9 indicates low impact; 1.9 < FI score ≤ 3.5 indicates medium impact; and FI score > 3.5 indicates high impact). Among the variants detected in exome sequencing from the tumor specimen, those in *SCN1B*, *PPP1R3A*, *GRID2*, *APBA2*, *ZNF142*, *ZYG11A*, *RBFOX1*, *TCF7L1*, *TEX13B*, and *DSCAML1* were validated by Sanger sequencing, and the details of these 10 candidate genes are provided in Table 2.

The primary tumor (sample #1), pleural metastases (sample #2), and malignant cells from ascites (sample #4) were all found to carry point mutations in *SCN1B*, *PPP1R3A*, and *ZYG11A*. However, *APBA2*, *ZNF142*, and *RBFOX1* mutations, although not present in the primary tumor, were detected in both metastatic (chemotherapy refractory) specimens. *TCF7L1*, *TEX13B*, and *DSCAML1* mutations, which were detected during exome sequencing, were not confirmed in subsequent Sanger sequencing of the primary tumor or any metastatic specimens. Although the mutation in *GRID2* was not seen in the primary tumor due to failure of the sequencing reactions, the mutation was confirmed in both metastatic samples.

### Comparative genomic hybridization array analysis of the primary tumor

**Figure 2:**
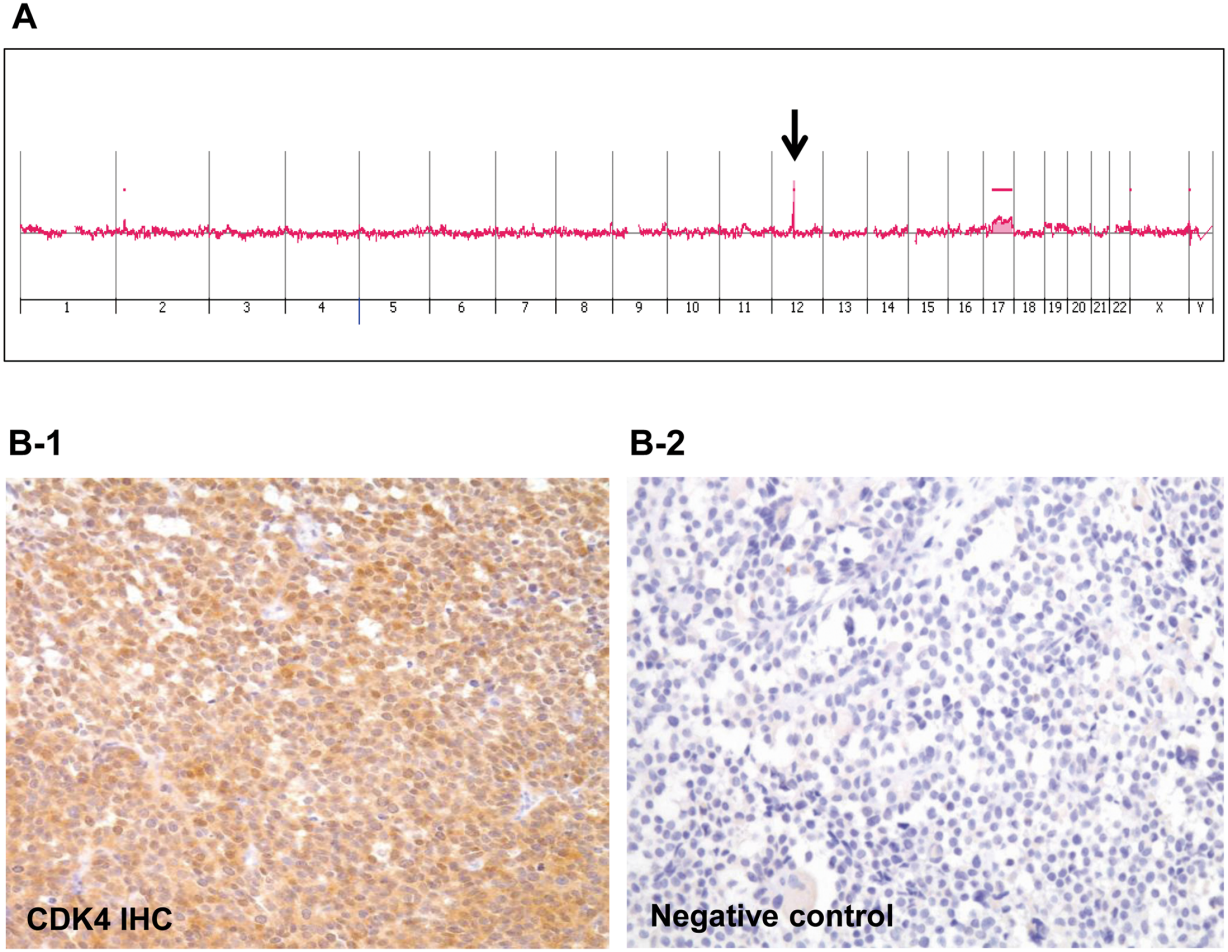
Array comparative genomic hybridization (aCGH) of the primary tumor and *CDK4* overexpression. (A). aCGH of the primary tumor: the 12q13.3–q14.1 region demonstrated the highest level of chromosomal amplification. (B-1). CDK4 immunohistochemistry of the primary tumor confirmed overexpression of CDK4 protein; *CDK4* is located within the 12q13.3-q14.1 region. (B-2). Negative control for CDK4 immunohistochemistry.

Although several chromosomal regions showed evidence of copy number variations (CNVs; Supplementary Table S1), the 12q13.3–q14.1 region demonstrated the highest level of chromosomal amplification (Fig. 2A). As this region contains multiple genes, we analyzed the amplification of each gene (Table 3). Within this region, the *CDK4* proto-oncogene was highly amplified, and several other genes in the amplicon also showed various degrees of amplification, including *NACA*, *HSD17B6*, *SDR9C7*, *RDH16*, *GPR182*, *ZBTB39*, *TAC3*, *MYO1A*, *NAB2*, *STAT6*, and *LRP1*. The overexpression of CDK4 at the protein level was also confirmed by IHC (Fig. 2B).

### ALK fusion assay

**Figure 3:**
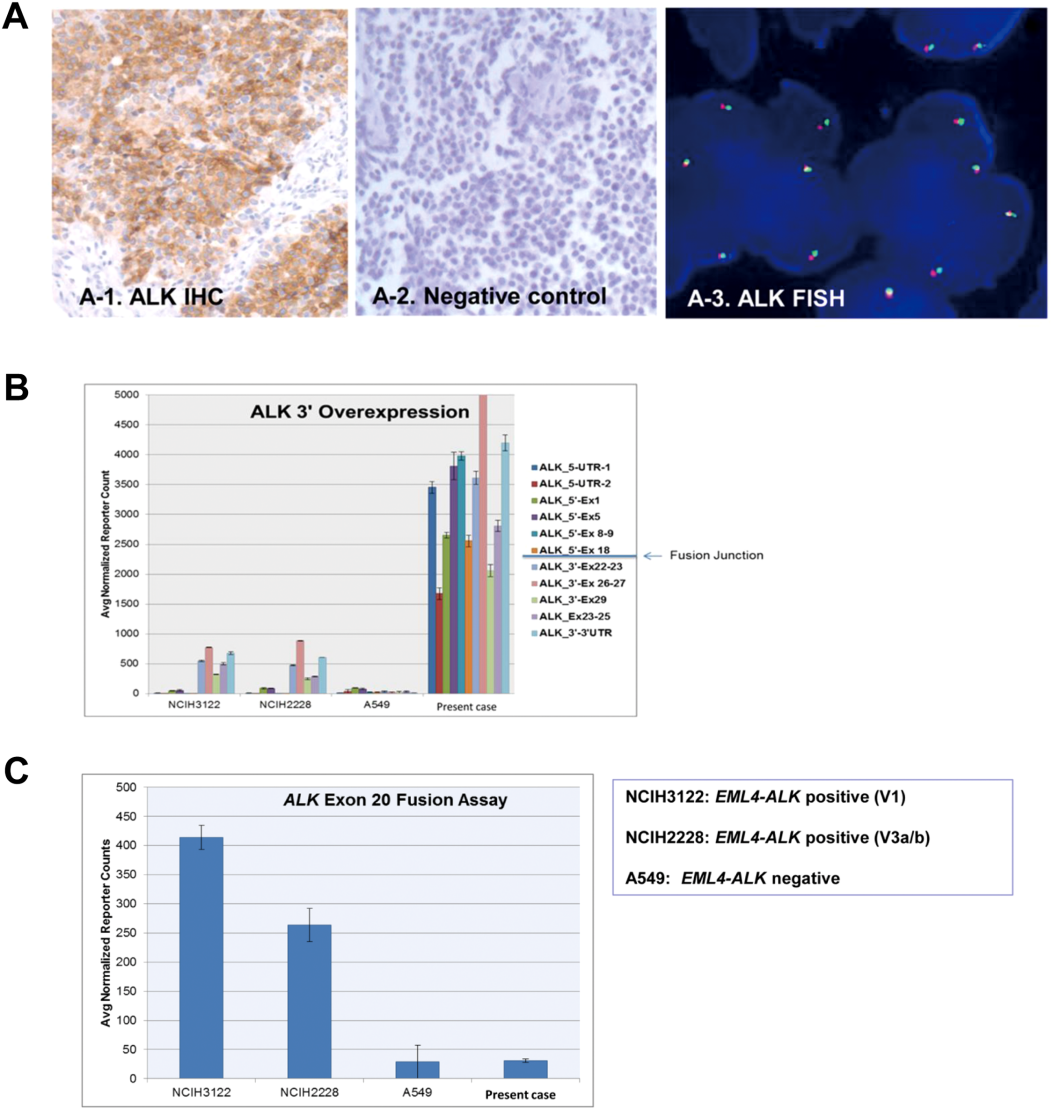
ALK expression and fusion assay. (A). ALK immunohistochemistry (2A-1, positive in the present case, 2A-2, negative control) and ALK FISH (2A-3) of the primary tumor: ALK protein overexpression was confirmed, but ALK rearrangement was not detected. (B). *ALK* RNA expression in tumor cells from pleural metastasis (sample#2): *ALK* RNA overexpression was detected. The *ALK* expression has been normalized to that of 4 housekeeping genes. Lung cancer cell lines (NCIH3122, NCIH2228, and A549) were used as controls for *ALK* RNA expression and *EML-ALK* fusion detection. (C). ALK fusion assay using NanoString: no *EML4-ALK* RNA was detected in the sarcoma specimen.

As *ALK* overexpression has been reported in refractory RMS^18^,^19^, we assessed the level of ALK protein expression using IHC. As shown in Fig. 3A-1, ALK IHC was strongly positive in most of the tumor cells. Given the high level of ALK protein expression, *ALK* RNA overexpression was also detected in the pleural metastasis specimen (sample #2), as expected (Fig. 3B).

Two lung cancer cell lines, NCIH3122 and NCIH2228, were used as positive controls for *EML4–ALK* fusion and A549 cells were used as the negative control. ALK-fusion lung cancer only overexpresses the 3′-*ALK* mRNA (NCIH3122 and NCIH2228), whereas sarcoma overexpresses the full-length mRNA. The mean of 3′-*ALK* expression of ALK^+^ lung tumor is approximately 500, whereas this reached approximately 3000 in the sarcoma. Despite *ALK* overexpression at the protein and RNA levels, *ALK* amplification was not observed in the tumor cells of this patient.

Next, we screened for the presence of *ALK* fusion partners using a NanoString assay; however, the *ALK* fusion assay failed to show any *ALK* rearrangements (Fig. 3C). *ALK* rearrangement was also not detected by FISH (Fig. 3A-3).

## Discussion

Patients with recurrent RMS usually present with a rapidly deteriorating condition and have markedly limited options in terms of chemotherapy^9^,^20^. In this study, we found that the majority of somatic mutations found during exome sequencing of primary tumor tissue were also observed in metastatic tumor tissue and metastatic cells in ascites samples, although Sanger sequencing revealed genetic alterations involving several genes, such as *APBA2*, *ZNF1142*, and *RBFOX1*, only in the metastatic samples. These results led to 2 important conclusions: First, there is little genetic heterogeneity between primary and metastatic RMS cells at least in terms of mutational spectra, reflecting relatively little genetic evolution during the course of metastasis. This is consistent with the results of recent similar studies on melanoma^21^, breast^22^, and pancreatic cancers^23^, in which genomic profiling for both primary and metastatic sites was performed. Second, we confirmed that malignant cells isolated from body fluid can be used for genomic profiling, as their genome is nearly identical to that of the resected tumor specimen. This may be especially important in clinical practice because body fluid can be obtained relatively easily using a bedside procedure. Of the 23 candidate genes found during exome sequencing, we selected 9 mutated genes (*APBA2*, *RBFOX1*, *TCF7L1*, *MUC16*, *UBA1*, *FEN1*, *NBEAL1*, *DSCAML1*, and *PCDHA4*) for which substantial functional impact was predicted (medium or high functional impact; FI score > 1.9), with or without damaging/deleterious phenotypic effects based on the SIFT score (≤0.05), and assessed their clinical relevance to RMS. However, we could not find any pre-existing evidence that these genetic alterations contribute to RMS development.

The aCGH array used in this study revealed prominent amplification in the 12q13 and 12q14 regions. Although we found that many genes within this chromosome 12 region were amplified, *CDK4* amplification was of particular interest because it is known to play a pivotal role in the oncogenic process^24^, and perhaps more importantly, the corresponding proteins are potential drug targets^25^. Its overexpression is frequently observed in well-differentiated and dedifferentiated liposarcomas^26^,^27^,^28^; consequently, a clinical trial of the CDK4 inhibitor (PD0332991) for *CDK4*-amplified tumors has been conducted. In both phase I and phase II trials, the CDK4 inhibitor has proven effective in C, and a randomized phase 3 trial is being considered by researchers^29^. Amplification of 12q13–q14 and *CDK4* in RMS has been reported previously^30^,^31^, as has amplification of *MYCN*, and both of these genes are known to be involved in RMS tumorigenesis^32^. However, these genes are associated with distinct expression profiles and clinical parameters. *MYCN* overexpression occurs more frequently in cases in which 2p24 amplification is present, whereas *CDK4* overexpression is associated with 12q13–14 amplification^33^. In addition, 12q13–14 amplification was significantly associated with poor clinical outcomes, such as short failure-free and overall survival, compared to that seen in cases with 2q24 amplification^33^. In the RMS case studied here, we confirmed the presence of a 12q13–14 amplification and showed that *CDK4* is one of the genes overexpressed in this chromosomal region.

A study provided *in vitro* evidence for the successful pharmacologic inhibition of CDK4/CDK6 activity in myoblasts and RMS-derived cells^34^; in this study, most ARMS-and ERMS-derived cell lines and tumor samples expressed CDK4 and *CDK6*, and exposure of these cells to a CDK4 inhibitor caused G1 cell cycle arrest, which is closely associated with myogenic differentiation. Given that defective cell cycle control, which leads to failure of myogenic differentiation, is one of the notable characteristics of RMS-derived cells, it was not surprising that CDK4 inhibition with PD0332991 ultimately facilitated skeletal muscle differentiation. This finding suggests that CDK4 inhibition is a potential therapeutic strategy for RMS. However, there is a scarcity of data on the use of a CDK4 inhibitor in patients with RMS. Although it is therefore difficult to draw firm conclusions regarding the potential efficacy of this inhibitor, the need for novel therapeutics arising from the dismal prognosis in refractory RMS, together with the genetic profiling data presented here, warrant clinical trials on a CDK4 inhibitor in chemotherapy-refractory RMS patients.

In agreement with previous reports^18^,^19^, we found that *ALK* was overexpressed in the RMS tumor in the current case. Although *ALK* overexpression is frequently detected in RMS, the mechanisms underlying this phenomenon are yet to be defined. However, a high-affinity binding site for the PAX3 and FOXO1 transcription factors in the intron of *ALK* has been reported to mediate high levels of *ALK* transcription^35^, and increases in *ALK* copy number have also been described^17^,^18^, although this did not always correlate with elevated ALK protein expression. A recent extensive cohort study on *ALK* aberration in RMS^17^ revealed that approximately 90% of ARMS patients and 50% of ERMS patients exhibited *ALK* copy number gains, whereas only 4% of RMS patients showed true amplification of *ALK*. In our study, *ALK* amplification was not observed, although *ALK* was overexpressed. The results of our study indicate that the overexpression of wild-type ALK alone may not be sufficient to drive tumor growth and that *ALK* may therefore not be an effective drug target in RMS. Currently, clinical trial NCT # 01121588 (clinicaltrials.gov) on crizotinib therapy for ALK-positive solid tumor types is ongoing. Since our study is limited to one case only, further studies are required to elucidate the antitumor efficacy of crizotinib and the CDK4 inhibitor in sarcomas in the context of clinical trials.

In summary, our study revealed that there was little genetic heterogeneity between primary and metastatic RMS cells and suggested that malignant cells from body fluid can be used for genomic profiling of RMS patients. The RMS tumor in this case overexpressed *ALK*, but this was not associated with the amplification or translocation of this gene. Prominent amplification of the 12q13–14 region was also observed, and we propose that *CDK4*, located in 12q14, is a potential target for drugs in RMS.

## Methods

### Ethics statement

This study was approved by the SMC Institutional Review Board and was conducted in accordance with the 1996 Declaration of Helsinki. Written informed consent was obtained from the patient before genomic analyses were performed for research purposes.

### IHC

Five-micrometer-thick tissue sections were deparaffinized in xylene, rehydrated, and heated to 100°C in citrate buffer (pH 6.0) for 5 min for non-enzymatic antigen retrieval. The sections were incubated with monoclonal mouse anti-human desmin antibodies (1:100 dilution; RLM30; Novocastra, Newcastle-upon-Tyne, UK) for 60 min at room temperature, followed by incubation with a 1:1000 dilution of biotinylated goat anti-mouse IgG (Vector Laboratories, Burlingame, CA, USA) for 1 h at room temperature. The sections were stained with diaminobenzidine chromogen for 5–10 min and were then counterstained with hematoxylin for 5 min.

### FISH

FISH was performed using commercially available *ALK* (Vysis LSI ALK Dual Color, Break Apart Rearrangement Probe; Abbott Molecular, Abbott Park, IL) and *FKHR* (Vysis LSI *FKHR* Dual Color, Break Apart Rearrangement Probe; Abbott Molecular) probes according to the manufacturer's instructions. One hundred cells were analyzed in each case. FISH was considered positive when more than 15% of the tumor cells showed distinct red and green signals and/or a single red (residual 3′) signal; alternatively, the specimen was classified as FISH negative.

### Biospecimen processing and quality control

Excised tumor tissues were divided into 2 pieces. One piece was embedded in optimal cutting temperature compound and used to prepare hematoxylin and eosin-stained frozen section slides. The other pieces of tissue were snap frozen in liquid nitrogen and stored at −80°C. The tumor cell populations on the frozen section slide accounted for more than 60% of the total cell population; less than 10% were necrotic. Genomic DNA was extracted from snap frozen tissue and peripheral blood using the QIAmp DNA Mini kit (Qiagen GmbH, Hilden Germany) according to the manufacturer's instructions. DNA integrity was evaluated using 1% agarose gel electrophoresis. Tumor and normal DNA concentrations were measured using PicoGreen dsDNA Quantitation Reagent (Invitrogen, Carlsbad, CA). A minimum DNA concentration of 20 ng/μl was required for aCGH.

### Exome sequencing and analysis

Genomic DNA was extracted from the blood and primary tumor (abdomen) of the RMS patient. Exon capture was performed using Agilent SureSelectXT Human All Exon (50 M), which includes all exons annotated in the consensus CDS (CCDS) database, as well as 10 bp of flanking sequence for each targeted region (http://www.genomics.agilent.com). The captured DNA fragments were sequenced with Illumine Hiseq2000, generating 100 bp × 2 paired-end reads. The clean reads were aligned against the human reference genome (hg19/GRCh37) using the Burrows-Wheeler Aligner (BWA). The alignment results were further processed sequentially using local realignment, duplicate read marking, and base quality recalibration by using the Picard (http://picard.sourceforge.net) and GATK (http://www.broadinstitute.org/gatk/) pipeline software. Variant and germline calling were performed using JointSNVMix (http://code.google.com/p/joint-snv-mix/), and the somatic mutations observed in tumor tissue were annotated using ANNOVAR (http://www.openbioinformatics.org/annovar/).

### Quality control, sequence alignment, somatic variant calling, and annotation

In the first quality control step, Cutadapt v.1.0 [1] removed adapter sequences from the input fastq sequence. After adapter trimming, Fastx v.0.0.13 [2] filtered low-quality reads, such that base quality was more than 20 and the proportion of good-quality bases in each read was more than 50%. Finally, cmpFastq [3] classified paired-end reads and single-end reads. Classified fastq sequences were aligned to the human reference sequence (hg19) using the Burrow-Wheeler Aligner v.0.5.9 (BWA) [4], and were then merged to a BAM file. Subsequently, sequential cleanup processes, consisting of the addition or replacement of read groups, marking and removing duplicates, and fixing mate information were performed using Picard Tools v.1.69 [5]. The cleaned bam file was then sorted using Samtools v.0.1.18 [6] and the local realignment and base quality score recalibration were processed using the Genome Analysis Toolkit v.1.6–7 (GATK) [7].

Somatic mutations were designated into 3 categories: single nucleotide polymorphisms (SNPs), indels, and CNVs. We began by applying the joint_snv_mix_one model in JointSNVMix v.0.7.5 [8] in order to find point mutations, and used Annovar [9], Mutation Assessor [10], and SIFT [11] for annotation. Annovar performed filter-based annotation indicating mutations that are present in 1000 genome projects or dbSNP (snp135). It also performed gene-based annotation using Mutation Assessor and SIFT to identify whether protein-coding changes caused by SNPs or CNVs are deleterious. We selected genes that were annotated as “medium or high functional impact” by Mutation Assessor and were predicted as “damaging” by SIFT. Indels were detected by the SomaticIndelDetector in GATK, following which Annovar gene-based annotation was used to describe the functional impact of somatic indels. CNVs were detected using ExomeCNV (R package) from the coverage file prepared using DepthOfCoverage in GATK. We used default parameters, except for the aforementioned software.

### aCGH

Genomic DNA was extracted from the cells cultured from the primary tumor of the patient. aCGH was performed using the Agilent Human Genome CGH Microarray Kit 8 × 60 K, which contains approximately 45,000 probes.

### ALK fusion transcript assay

nCounter assays were performed in duplicate, according to the manufacturer's instructions (NanoString Technologies, Inc, Seattle, WA, USA). Briefly, 500 ng of total RNA was hybridized to nCounter probe sets for 16 h at 65°C. Samples were processed using an automated nCounter Sample Prep Station (NanoString Technologies, Inc). Cartridges containing immobilized and aligned reporter complexes were subsequently imaged on the nCounter Digital Analyzer (NanoString Technologies, Inc), set at 1155 fields of view. Reporter counts were collected using the nSolver analysis software version 1 in NanoString, normalized, and analyzed as described below. A detailed description of the assay is given elsewhere^36^.

## Author Contributions

J.L. and Y.C. conceived the idea and designed the study. S.P. and J.L. collected and analyzed data, and S.P. and I.D. wrote the main manuscript text. Y.C., I.D. and M.M. prepared Figures 2 and 3. J.J., K.R., S.A., L.M., S.K., K.K., M.M., J.K., E.O. and Y.K. contributed by providing study material. All authors reviewed the manuscript.

## Supplementary Material

Supplementary Information Supplementary Information

## Figures and Tables

**Table 1:** Somatic variants detected in rhabdomyosarcoma (RMS; primary tumor, sample #1) by whole exome sequencing

No	Ch	position	gene name	ref	var	vac	tr	vrf	vac	tr	vrf	func. Impact	fi score	prediction	score
1	19	35524413	SCN1B	A	G	0	35	0.0%	11	21	52.4%	low	1.865	D	0
2	7	113519206	PPP1R3A	A	T	0	102	0.0%	66	142	46.5%	neutral	0	D	0.03
3	4	94411875	GRID2	C	A	0	61	0.0%	31	67	46.3%			D	0
4	15	29346353	APBA2	G	A	0	62	0.0%	23	53	43.4%	medium	2.28	D	0.02
5	2	219513493	ZNF142	C	T	0	24	0.0%	9	21	42.9%	low	0.875	T	0.3
6	1	53347155	ZYG11A	G	T	0	53	0.0%	18	43	41.9%			D	0
7	16	7568302	RBFOX1	C	A	0	74	0.0%	37	93	39.8%	medium	2.595	D	0
8	2	85533345	TCF7L1	G	A	0	23	0.0%	6	19	31.6%	medium	2.25	T	0.05
9	17	80445942	NARF	A	G	0	23	0.0%	8	28	28.6%	neutral	0.455	D	0.03
10	16	85698723	KIAA0182	T	A	0	24	0.0%	4	16	25.0%	low	1.32	D	0.01
11	X	107224952	TEX13B	G	A	0	47	0.0%	12	54	22.2%	low	1.5	T	0.06
12	11	1093375	MUC2	C	T	0	26	0.0%	4	18	22.2%			T	0.23
13	21	45877015	LRRC3	A	C	0	28	0.0%	4	20	20.0%	low	0.865	T	0.11
14	1	24663626	GRHL3	A	C	0	26	0.0%	4	22	18.2%	low	1.67	D	0
15	19	9005714	MUC16	A	C	2	58	3.4%	8	44	18.2%	medium	1.935	T	0.13
16	18	29178556	TTR	G	C	0	28	0.0%	4	23	17.4%	low	1.545	D	0
17	X	47069419	UBA1	G	C	0	41	0.0%	6	36	16.7%	medium	3.34	D	0
18	11	61563225	FEN1	T	G	0	29	0.0%	5	32	15.6%	medium	2.83	D	0
19	17	12896247	ELAC2	A	C	0	26	0.0%	4	26	15.4%	low	1.87	D	0
20	2	204045181	NBEAL1	A	C	0	72	0.0%	9	66	13.6%	high	4.49	D	0
21	11	117342607	DSCAML1	A	C	0	37	0.0%	5	37	13.5%	medium	2.93	D	0
22	5	140188268	PCDHA4	T	G	0	39	0.0%	5	37	13.5%	medium	2.905	D	0
23	1	145601821	POLR3C	G	C	0	39	0.0%	5	37	13.5%	low	1.39	D	0.04

Ch, chromosome; ref, reference; var, variant; vac, variant allele count; tr, total read; vrf, variant read frequency; D, damaging; T, tolerated; SIFT, Sorting Tolerant From Intolerant (Nucleic Acids Res 2003;31:3812).

Nos. 1, 2, 3, 4, 5, 6, 7, 8, 11, and 21: somatic mutations that were validated by Sanger sequencing (Table 2).

No. not mentioned above: somatic mutations that were not validated by Sanger sequencing.

**Table 2:** Information on candidate genes and validation of somatic mutations by Sanger sequencing

		Exome sequencing (sample #1)		Sanger sequencing		
Gene Name	Gene Description	Allele (%)	Variant	Primary Tumor (sample #1)	Ascites (sample #4)	Pleural metastases (sample #2)
**SCN1B**	sodium channel, voltage-gated, type I, beta	52.4	c.218A > G:p.Y73C	218A > G	218A > G	218A > G
**PPP1R3A**	protein phosphatase 1, regulatory subunit 3A	46.5	c.1941T > A:p.D647E	1941T > A	1941T > A	1941T > A
**GRID2**	glutamate receptor, ionotropic, delta 2	46.3	c.1944C > A:p.Y648X	Fail	1944C > A	1944C > A
**APBA2**	amyloid beta (A4) precursor protein-binding, family A, member 2	43.4	c.266G > A:p.G89D	WT	266G > A	266G > A
**ZNF142**	zinc finger protein 142	42.9	c.1138G > A:p.A380T	WT	1138G > A	1138G > A
**ZYG11A**	zyg-11 homolog A (C. elegans)	41.9	c.1762G > T:p.E588X	1762G > T	1762G > T	1762G > T
**RBFOX1**	RNA binding protein, fox-1 homolog (C. elegans) 1	39.8	c.241C > A:p.H81N	WT	241C > A	241C > A
**TCF7L1**	transcription factor 7-like 1 (T-cell specific, HMG-box)	31.6	c.1006GA:p.V336M	WT	WT	WT
**TEX13B**	testis expressed 13B	22.2	c.406C > T:p.L136F	WT	WT	WT
**DSCAML1**	Down syndrome cell adhesion molecule like 1	13.5	c.3110T > G:p.L1037R	WT	WT	WT

**Table 3:** Amplification of genes within the 12q13.3–14.1 region

CytoBand	Start	Stop	Genes	Description	Logratio	Amplification
q13.3	57113710	57113768	**NACA**	nascent polypeptide-associated complex alpha subunit	3.08714845	1.9722129
q13.3	57163276	57163335	**HSD17B6**	hydroxysteroid (17-beta) dehydrogenase 6 homolog	2.4834826	1.9722129
q13.3	57320705	57320764	**SDR9C7**	short chain dehydrogenase/reductase family 9C, member 7	4.02499225	3.5578115
q13.3	57346683	57346741	**RDH16**	retinol dehydrogenase 16	3.5232894	3.5578115
q13.3	57388559	57388616	**GPR182**	G protein-coupled receptor 182	4.0685396	3.5578115
q13.3	57393071	57393130	**ZBTB39**	zinc finger and BTB domain containing 39	4.8680987	3.5578115
q13.3	57407139	57407192	**TAC3**	tachykinin 3	3.0193808	3.5578115
q13.3	57434942	57435000	**MYO1A**	myosin IA	3.6858533	3.5578115
q13.3	57485677	57485731	**NAB2**	NGFI-A binding protein 2 (EGR1 binding protein 2)	3.68784485	3.5578115
q13.3	57494332	57494389	**STAT6**	signal transducer and activator of transcription 6, interleukin-4 induced	3.0424881	3.5578115
q13.3	57536486	57536545	**LRP1**	low density lipoprotein receptor-related protein 1	3.46636355	3.5578115
q13.3	57627832	57627881	SHMT2	serine hydroxymethyltransferase 2	1.191406	1.9722129
q13.3	57631500	57631556	NDUFA4L2	NADH dehydrogenase	1.3683021	1.9722129
q13.3	57640585	57640634	STAC3	SH3 and cysteine rich domain 3	0.29862076	0.3607726
q13.3	57648401	57648459	R3HDM2	R3H domain containing 2	0.1239139	0.3607726
q13.3	57836402	57836461	INHBC	inhibin, beta C	−0.1627071	0.3607726
q13.3	57851519	57851578	INHBE	inhibin, beta E	0.14920259	0.3607726
q13.3	57856767	57856826	GLI1	GLI family zinc finger 1	1.0021597	0.3607726
q13.3	57866181	57866240	ARHGAP9	Rho GTPase activating protein 9	0.52336573	0.3607726
q13.3	57882471	57882530	MARS	methionyl-tRNA synthetase	0.62351316	0.3607726
q13.3	57911186	57911234	DDIT3	DNA-damage-inducible transcript 3	0.1061752	0.3607726
q13.3	57933393	57933452	DCTN2	dynactin 2 (p50)	0.8863068	0.3607726
q13.3	57948906	57948965	KIF5A	kinesin family member 5A	0.40449824	0.3607726
q13.3	57988882	57988939	PIP4K2C	phosphatidylinositol-5-phosphate 4-kinase, type II, gamma	0.23406802	0.3607726
q13.3	58000377	58000436	DTX3	deltex homolog 3 (Drosophila)	0.07017814	0.3607726
q13.3	58009250	58009302	GEFT	RhoA/RAC/CDC42 exchange factor	0.29716232	0.3607726
q13.3	58018895	58018942	SLC26A10	solute carrier family 26, member 10	0.89334315	0.3607726
q13.3	58025318	58025372	B4GALNT1	beta-1,4-N-acetyl-galactosaminyl transferase 1	0.54608015	0.3607726
q14.1	58110202	58110247	OS9	osteosarcoma amplified 9, endoplasmic reticulum lectin	2.8875618	3.2700999
q14.1	58118971	58119026	AGAP2	ArfGAP with GTPase domain, ankyrin repeat and PH domain 2	3.2052916	3.2700999
q14.1	58139960	58140017	TSPAN31	tetraspanin 31	2.3439372	3.2700999
q14.1	58143212	58143260	CDK4	cyclin-dependent kinase 4	3.8085285	3.2700999
q14.1	58163728	58163784	METTL1	methyltransferase like 1	3.1829998	3.2700999
q14.1	58167136	58167187	FAM119B	family with sequence similarity 119, member B	3.5654607	3.2700999
q14.1	58186836	58186894	TSFM	Ts translation elongation factor, mitochondrial	3.2734551	3.2700999
q14.1	58205712	58205771	AVIL	advillin	3.2119691	3.2700999

**Bold**: genes that were detected also in exome copy number variation (CNV).
