# Supplementary material for: Aberrant CDK4 Amplification in Refractory Rhabdomyosarcoma as Identified by Genomic Profiling
Source: Sci Rep. 2014 Jan 10;4:3623. doi: 10.1038/srep03623 (PMC3887377; doi:10.1038/srep03623)
Supplement: Supplementary Information [file srep03623-s1.pdf]

SUPPLEMENTARY INFORMATION

Aberrant CDK4 Amplification in Refractory Rhabdomyosarcoma as Identified by Genomic Profiling

Silvia Park[1], Jeeyun Lee[1*], In-Gu Do[2], Jiryeon Jang[2], Kyoohyoung Rho[3], Seonjoo Ahn[3], Lira Maruja[4], Sung Joo Kim[5], Kyoung-Mee Kim[2], Mao Mao[4], Jhingook Kim[6], Ensel Oh[7], Yu Jin Kim[7] & Yoon-La Choi[2*]

[1]Department of Medicine, Division of Hematology-Oncology, Samsung Medical Center, Sungkyunkwan University School of Medicine, Seoul, Korea, [2]Department of Pathology, Samsung Medical Center, Sungkyunkwan University School of Medicine, Seoul, Korea, [3]Korean Bioinformation Center (KOBIC), KRIBB, Daejeon 305-806, Korea, [4]Pfizer Oncology, 10724 Science Center Dr, San Diego, CA 92121, USA, [5]Department of Surgery, Samsung Medical Center, Sungkyunkwan University School of Medicine, Seoul, Korea, [6]Department of Thoracic Surgery, Samsung Medical Center, Sungkyunkwan University School of Medicine, Seoul, Korea, [7]Laboratory of Cancer Genomics and Molecular Pathology, Samsung Biomedical Research Institute, Samsung Medical Center, Seoul, Korea.

[*]**Co-Correspondences to**:

Jeeyun Lee, M.D., Ph.D.

Division of Hematology/Oncology, Department of Medicine

Samsung Medical Center, Sungkyunkwan University School of Medicine

50 Irwon-dong Gangnam-gu

Seoul 135-710 Korea

Tel: +82-2-3410-1779; Fax: +82-2-3410-1754

Email: jyunlee@skku.edu

Yoon-La Choi, M.D., Ph.D.

Department of Pathology

Samsung Medical Center, Sungkyunkwan University School of Medicine

50 Irwon-dong Gangnam-gu

Seoul 135-710 Korea

Tel: +82-2-3410-2797; Fax: +82-2-3410-

Email: ylachoi@skku.edu

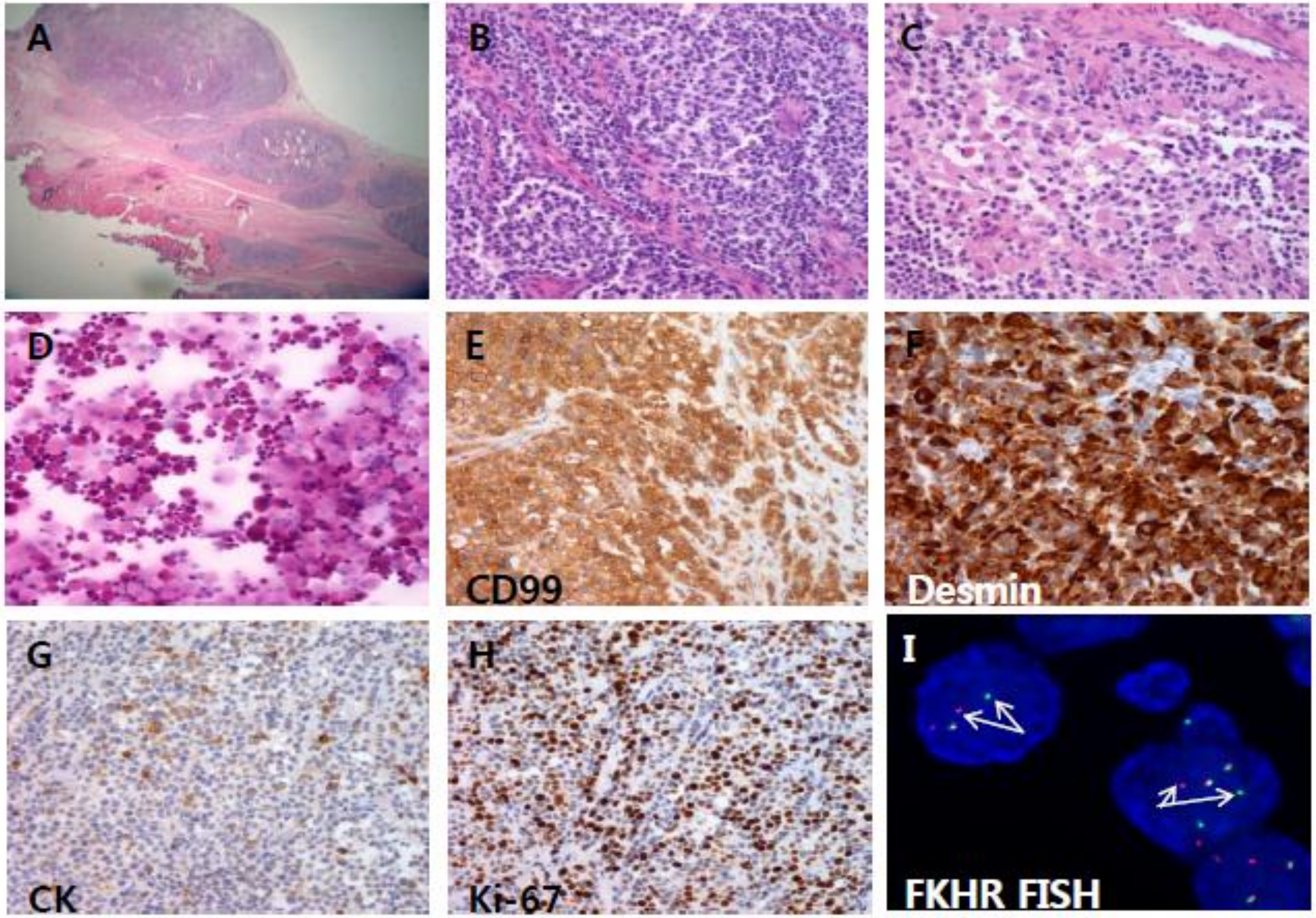

Figure S1. Pathology of primary tumor and the results of immunohistochemistry (IHC) and fluorescence in situ hybridization (FISH)

Table S1. Chromosomal amplification detected in array comparative genomic hybridization (CGH)

	Chr	Cytoband	Start	Stop	#Probes	Amplification	Deletion	pval	Gene Names
1	chr2	p24.1 - p23.3	23627041	25235327	36	0.78558	0	4.05E-29	KLHL29, ATAD2B, UBXN2A, MFSD2B, C2orf44, FKBP1B, SF3B14, TP53I3, PFN4, LOC375190, C2orf84, ITSN2, NCOA1, C2orf79, CENPO, ADCY3, DNAJC27
2	chr3	q26.31	172538344	172538403	5	1.502986	0	1.00E-15	ECT2
3	chr4	q28.1	124205362	124235444	6	0	-1.16656	7.38E-12	SPATA5
4	chr4	q34.1	173429497	173429556	5	0	-2.77025	7.85E-49	GALNTL6
5	chr6	q27	166579299	166593404	6	1.270815	0	9.58E-14	T
6	chr8	p23.1	11141188	11141247	5	1.593977	0	1.85E-17	
7	chr8	q24.3	142238251	145811230	101	0.524174	0	7.04E-36	SLC45A4, GPR20, PTP4A3, FLJ43860, NCRNA00051, TSNARE1, BAI1, ARC, JRK, PSCA, LY6K, C8orf55, SLURP1, LYPD2, LYNX1, LY6D, GML, CYP11B1, CYP11B2, LY6E, LY6H, GPIHBP1, ZFP41, GLI4, ZNF696, TOP1MT, RHPN1, MAFA, ZC3H3, GSDMD, NAPRT1, EEF1D, TIGD5, PYCRL, TSTA3, ZNF623, ZNF707, BREA2, MAPK15, FAM83H, SCRIB, MIR937, PUF60, NRBP2, EPPK1, PLEC1, MIR661, PARP10, GRINA, SPATC1, OPLAH, EXOSC4, GPAA1, CYC1,

									SHARPIN, MAF1, KIAA1875, HEATR7A, SCXB, SCXA, BOP1, HSF1, DGAT1, SCRT1, C8ORFK29, FBXL6, GPR172A, ADCK5, CPSF1, MIR939, MIR1234, SLC39A4, VPS28, NFKBIL2, CYHR1, KIFC2, FOXH1, PPP1R16A, GPT, MFSD3, RECQL4, LRRC14, LRRC24, MGC70857,
8	chr11	p13	35269915	35276999	6	1.284579	0	5.26E-14	SLC1A2
9	chr12	q13.3 - q14.1	57113710	58205771	76	1.972213	0	0	NACA, PRIM1, HSD17B6, SDR9C7, RDH16, GPR182, ZBTB39, TAC3, MYO1A, TMEM194A, NAB2, STAT6, LRP1, MIR1228, NXPH4, SHMT2, NDUFA4L2, STAC3, R3HDM2, INHBC, INHBE, GLI1, ARHGAP9, MARS, DDIT3, MBD6, DCTN2, KIF5A, PIP4K2C, DTX3, GEFT, SLC26A10, B4GALNT1, OS9, AGAP2, LOC100130776, TSPAN31, CDK4, MARCH9, CYP27B1, METTL1, FAM119B, TSFM, AVIL
10	chr12	q13.3	57211488	57548099	17	3.557811	0	2.87E-52	SDR9C7, RDH16, GPR182, ZBTB39, TAC3, MYO1A, TMEM194A, NAB2, STAT6, LRP1
11	chr12	q13.3	57640585	58025372	31	0.360773	0	4.34E-100	STAC3, R3HDM2, INHBC, INHBE, GLI1, ARHGAP9, MARS, DDIT3, MBD6, DCTN2, KIF5A, PIP4K2C, DTX3, GEFT, SLC26A10, B4GALNT1
12	chr12	q13.3 - q14.1	58087409	58205771	15	3.2701	0	1.06E-32	OS9, AGAP2, LOC100130776, TSPAN31, CDK4,

									MARCH9, CYP27B1, METTL1, FAM119B, TSFM, AVIL
13	chr14	q24.3	77234043	77599248	23	1.008947	0	1.40E-30	VASH1, ANGEL1, ZDHHC22
14	chr17	q11.2 - q25.3	26727329	80969424	1623	0.688719	0	0	
15	chrX	p22.33	169064	2778548	94	0.559882	0	5.67E-38	PLCXD1, GTPBP6, NCRNA00107, PPP2R3B, SHOX, CRLF2, CSF2RA, IL3RA, SLC25A6, NCRNA00105, ASMTL, P2RY8, SFRS17A, ASMT, DHRSX, ZBED1, CD99, XG, XGPY2, GYG2
16	chrX	p22.11	23858412	23858471	5	1.373503	0	1.99E-13	APOO
17	chrX	q28	152485412	154194423	64	0.560647	0	1.50E-26	MAGEA1, ZNF275, ZFP92, TREX2, HAUS7, BGN, ATP2B3, FAM58A, DUSP9, PNCK, SLC6A8, BCAP31, ABCD1, PLXNB3, SRPK3, IDH3G, SSR4, PDZD4, L1CAM, AVPR2, ARHGAP4, NAA10, RENBP, HCFC1, TMEM187, IRAK1, MIR718, MECP2, OPN1LW, OPN1MW, OPN1MW2, TEX28, TKTL1, FLNA, EMD, RPL10, SNORA70, DNASE1L1, TAZ, ATP6AP1, GDI1, FAM50A, PLXNA3, LAGE3, UBL4A, SLC10A3, FAM3A, G6PD, IKBKG, CTAG1B, CTAG1A, CTAG2, GAB3, DKC1, SNORA36A, SNORA56, MPP1, F8, H2AFB3, H2AFB1, F8A1, F8A2, F8A3
18	chrY	p11.32 - p11.31	119064	2606392	91	0.564644	0	2.03E-37	PLCXD1, GTPBP6, PPP2R3B, SHOX, CRLF2, CSF2RA, IL3RA, SLC25A6, NCRNA00105, ASMTL, P2RY8,

	SFRS17A, ASMT, DHRSX, ZBED1, CD99
